# Protein S Deficiency with Recurrent Deep Vein Thrombosis and Post Thrombotic Syndrome: A Case Report

**DOI:** 10.31729/jnma.7694

**Published:** 2022-10-31

**Authors:** Shashank Neupane, Prasamsa Pudasaini, Bishal Dhakal, Shila Awal, Sajeena Thapa, Binaya Subedi

**Affiliations:** 1 Nepalese Army Institute of Health Sciences, Sanobharyang, Kathmandu, Nepal; 2 Department of Internal Medicine, Nepalese Army Institute of Health Sciences, Sanobharyang, Kathmandu, Nepal

**Keywords:** *deep vein thrombosis*, *protein S deficiency*, *rivaroxaban*

## Abstract

Protein S is a vitamin K-dependent protein that acts as a break in secondary hemostasis by inactivating activated factor V and activated factor VIII. We report a case of a 40 years old male who had the first episode of deep vein thrombosis of the left lower limb 10 years back, which despite treatment, reoccurred 3 months later in the bilateral lower limb. Thrombophilic screening showed severe protein S deficiency. The patient then developed deep vein thrombosis of both upper limbs. The patient was advised to place an inferior vena cava filter, which he denied. The patient is now presenting with multiple episodes of post-thrombotic syndrome. Such attacks are treated with elastic compression stockings, rivaroxaban, and morphine. However, despite medication, the pain has not yet subsided. Hence, even though protein S deficiency is the rare cause of deep vein thrombosis when recurrent should be considered despite its rare occurrence.

## INTRODUCTION

Protein S, a cofactor of protein C, operates as an inhibitor of factors Va and VIIIa.^1^ Protein S deficiency is an autosomal dominant inherited coagulation disorder. However, acquired Protein S deficiency occurs secondary to liver disorders, malignancy, pregnancy, nephrotic syndrome, and acute phase reactions.^2^ Protein S is principally synthesized in hepatocytes and ordinarily in megakaryocytes, osteoblasts, endothelial, Leydig, and vascular smooth muscle cells.^3^ The prevalence of protein S deficiency is 0.03-0.13% in Western countries.^1^ Here, we present a case of 40 years old male with protein S deficiency leading to chronic recurrent deep vein thrombosis (DVT) presenting with multiple episodes of post-thrombotic syndrome.

## CASE REPORT

Forty-year-old male with no known comorbidities started having muscle cramps involving bilateral lower limbs early morning ten years back, which continued for a week. The patient was advised to drink plenty of water, suspecting dehydration. However, the symptoms did not resolve despite adequate hydration. The patient then experienced painful swelling over the left lower limb, which started in the calf region and grew upward towards the thigh. There was no history of similar illness or recent trauma or any major surgical procedure. Venous doppler showed hypoechoic thrombus in the lumen of the left external iliac and superficial femoral veins with incompressibility and complete occlusion of veins. Echocardiogram showed normal scan. Warfarin 5 mg once daily was initiated on day 2 of unfractionated heparin therapy. Warfarin and parenteral anticoagulants overlapped for 5 days then heparin was discontinued. Warfarin was prescribed for three months along with morphine for pain management.

Three months later, the patient had a second episode of DVT involving bilateral lower limbs. Thrombophilic screening showed low protein S levels of 22% performed using Enzyme Linked Immuno Sorbent Assay (ELISA). Protein C level was normal (i.e. 123%) done via the chromogenic method. To rule out hepatic dysfunction, nephrotic syndrome, malignancy, and acute-phase reaction as the acquired cause of protein S deficiency; liver function test, urinary albumin creatinine ratio (ACR), serum ferritin, and contrast-enhanced Computed Tomography (CECT) scan (i.e. chest, abdomen, and pelvis) were sent which turned out to be normal. For an expert opinion, the patient went to India, where the patient started developing similar symptoms of DVT in the left upper limb, and a Duplex scan was used for diagnosis.

The patient was admitted to the same hospital for 54 days; on the 30^!th^ day, the patient also developed DVT of the right upper limb. Soon, experts advised the placement of IVC filters, keeping in mind the rapid progression of disease and possibilities of life-threatening pulmonary embolism. However, after explaining the risk and potential complications, the patient refused the procedure.

The patient had developed multiple episodes of symptoms of the post-thrombotic syndrome, such as pain, swelling of limbs, heaviness, and pruritus, which showed diurnal variation worsening at the end of the day where Color Doppler Flow Imaging (CDFI) showed chronic DVT changes with complete recanalisation of vessels. Treatment of such episodes with elastic compression stockings (ECS), rivaroxaban (i.e., factor X inhibitor) 20 mg per oral daily for 6 months, anti-oxidants (i.e., Vitamin E 400 mg once daily), and morphine (i.e., pain management) per oral 10 mg. However, despite medication, the patient has been having continuous pain in the upper and lower limbs asymmetrically. Advice for catheter-directed techniques was given, but the patient refused all invasive interventions. The patient is under regular follow-ups where a thorough examination along with a doppler study is performed to assess the recurrence of the disease. However, recently a patient was admitted to the ward due to morphine withdrawal as an adverse event of drug therapy.

## DISCUSSION

Protein S was first isolated in 1977, and since then, it has been proposed to have critical functions in various processes.^4^ Protein S plays a paramount and multifunctional function in regulating clot formation. Compared to most other vitamin K-dependent plasma proteins, Protein S is not a serine protease enzyme, but functions as a cofactor in the protein C regulatory system.^5^ Protein S deficiency commonly leads to recurrent deep vein thrombosis and pulmonary embolism; however, involvement of superficial, cerebral, visceral or axillary veins is less likely. Approximately half of the thromboembolic events are unprovoked, i.e., not preceded by transient risk factors such as surgery, trauma, immobilization, air travel, and hormone replacement therapy.^3^ Given this statement, our patient also did not have any risk factors to provoke DVT.

Winter peak for DVT demonstrates circannual rhythm in patients with protein C and S deficits.^6^ Similarly, in our patient total of three episodes of DVT were during the winter season. This result can be explained by the changes in the blood flow with characteristically increased red blood cells numbers, resulting in increased blood viscosity and a decrease in antithrombin III.^6^

Low-risk patients (e.g., patients without active cancer, outpatients, and patients without previous venous thromboembolic disease (VTE) with symptomatic distal DVT may benefit more from elastic compression stockings and ultrasound monitoring than therapeutic anticoagulant treatment.^7^ However, our patient had DVT involving both the distal and proximal veins. DVT is diagnosed with increasing precision using an expanding array of imaging modalities, including CDFI, computed tomography (CT), and magnetic resonance (MR) venography. DVT treatment has traditionally included Vitamin K antagonists such as Warfarin with Heparin. With the arrival of Direct Oral Anticoagulants came hope for more therapeutic options for DVT, but these newer agents' safety and efficacy profile compared to conventional therapy has been of paramount importance. Although the safety profile for direct oral anticoagulants (DOACs) has been quite favourable, dabigatran and rivaroxaban have increased GI bleeding.^8^

American Heart Association (AHA), American College of Chest Physicians (ACCP), and American College of Radiology (ACR)/Society of Interventional Radiology (SIR) advise the use of inferior vena cava (IVC) filters in patients that have the VTE with an absolute contraindication to anticoagulation, complications necessitating termination of anticoagulation, or who have recurrent VTE despite adequate anticoagulation.^9^ Similarly, our patient was advised to use an IVC filter, but the patient refused all kinds of invasive procedures.

The patient started having Post-Thrombotic Syndrome (PTS) symptoms secondary to prior DVT. PTS is a form of chronic venous insufficiency that affects up to 50% of patients after proximal DVT. There is no effective treatment of established PTS; however, prevention after DVT is possible. Anticoagulation is essential for PTS prevention. Among anticoagulants, low-molecular-weight heparins have anti-inflammatory properties. ECS may help treat acute DVT symptoms, but their benefits for PTS prevention are debated. Catheter-directed techniques reduce acute DVT symptoms and might reduce the risk of moderate-severe PTS in the long term in patients with iliofemoral DVT at low risk of bleeding.^10^ Our patient is treated with elastic compression stockings (ECS), rivaroxaban (i.e. factor X inhibitor), anti-oxidants, and morphine (i.e. for pain management). The patient is well adherent to medications, however, refuses all invasive procedures when the risk and benefits were explained. In light of the cure, the patient is still wandering to many clinicians however the actual cure is still not possible.

Though protein S deficiency can commonly lead to DVT, its occurrence as a cause for recurrent DVT is a rare one as we have presented here. Likewise, regular follow-up with the patient for clinical assessment represents the strength of our case study. However, there were certain limitations worth describing in our report. We could not convince an IVC to filter the patient which was an ideal treatment strategy in our case. Despite our full effort, we could not obtain a few hospital records which were damaged and lost during an earthquake in Nepal.

In conclusion, it is equally important to consider protein S deficiency as a rare but important cause of deep vein thrombosis. Clinicians should take it into consideration as the chances of missing the cause is more likely in low-resource countries. As it is a life-threatening recurring condition, adherence to medication and regular follow-ups are mandatory. Proper management can increase the quality of life in patients, however, despite which patients can have adverse events following various medications.

## References

[1] Komwilaisak P, Sasanakul W, Chuansumrit A, Kanjanapongkul S, Wangruangsathit S, Lusawat A (2017). Genotypes and phenotypes of protein S deficiency in Thai children with thromboembolism.. Pediatr Blood Cancer..

[2] Joshi A, Jaiswal JP (2010). Deep vein thrombosis in protein S deficiency.. J Nepal Med Assoc..

[3] Ten Kate MK, van der Meer J (2008). Protein S deficiency: a clinical perspective.. Haemophilia..

[4] Gierula M, Ahnstrom J (2020). Anticoagulant protein S-new insights on interactions and functions.. J Thromb Haemost..

[5] Marlar RA, Gausman JN (2011). Protein S abnormalities: a diagnostic nightmare.. Am J Hematol..

[6] Bilora F, Boccioletti V, Manfredini E, Petrobelli F, Tormene D, Simioni P (2002). Seasonal variation in the incidence of deep vein thrombosis in patients with deficiency of protein C or protein S.. Clin Appl Thromb Hemost..

[7] Robert-Ebadi H, Righini M (2017). Management of distal deep vein thrombosis.. Thromb Res..

[8] Stone J, Hangge P, Albadawi H, Wallace A, Shamoun F, Knuttien MG (2017). Deep vein thrombosis: pathogenesis, diagnosis, and medical management.. Cardiovasc Diagn Ther..

[9] Muneeb A, Dhamoon AS (2022). Inferior vena cava filter [Internet]..

[10] Makedonov I, Kahn SR, Galanaud JP (2020). Prevention and management of the post-thrombotic syndrome.. J Clin Med..

